# Endovascular Retrieval of a Fractured Tunneled Hemodialysis Central Venous Catheter Using the Loop Snare Technique

**DOI:** 10.7759/cureus.35617

**Published:** 2023-02-28

**Authors:** Tomoko Sasaki, Yuki Fujioka, Haruka Hikichi, Daisuke Yokota, Shigeharu Ueki

**Affiliations:** 1 Department of General Internal Medicine and Clinical Laboratory Medicine, Akita University Graduate School of Medicine, Akita, JPN; 2 Department of Hematology, Nephrology, and Rheumatology, Akita University Graduate School of Medicine, Akita, JPN; 3 Division of Cardiology, Ritsuzankai Iida Hospital, Iida, JPN

**Keywords:** loop snare, tunneled dialysis catheter, fluoroscopy intervention, foreign body removal, percutaneous cardiac intervention

## Abstract

The tunneled cuffed hemodialysis catheter is a valuable vascular access option for patients with end-stage renal disease (ESRD). Healthcare providers have become more familiar with the insertion of medical devices, including central venous catheters, in their daily practice. The occurrence of foreign body fragmentation is rare with these catheters. This article presents a case in which a fracture of the distal portion of the hemodialysis catheter was inadvertently identified during a coronary angiography. Percutaneous removal of the fractured venous catheter was performed successfully using a loop snare catheter, which prevented the patient from experiencing further complications.

## Introduction

Permanent vascular access is necessary for patients undergoing hemodialysis to achieve long-term dialysis stabilization. Autogenous arteriovenous fistula (AVF) is the most common and ideal vascular access because of its high patency rate and low risk of mortality [1]. However, AVF creation is difficult in patients with low ejection fraction, limited surface blood vessels, and arterial occlusion. For these patients, the tunneled cuffed hemodialysis catheter is a useful alternative to performing dialysis, regardless of its association with significant morbidity and high complication rates [2]. Inserting foreign bodies, such as central venous catheters, carries a high risk of complications. Among complications associated with central venous catheter insertion, catheter fracture accounts for only 0.1% of all cases [3]. Therefore, it is rare for interventionists to perform the retrieval of a fractured fragment. Here, we report a case in which the distal portion of the inserted venous catheter was fractured and was successfully removed using the endovascular approach.

This clinical case was previously presented as an oral presentation at the 24th Cardiovascular Summit Transcatheter Cardiovascular Therapeutics Asia Pacific (TCTAP) on April 29, 2019.

## Case presentation

**Figure 1:**
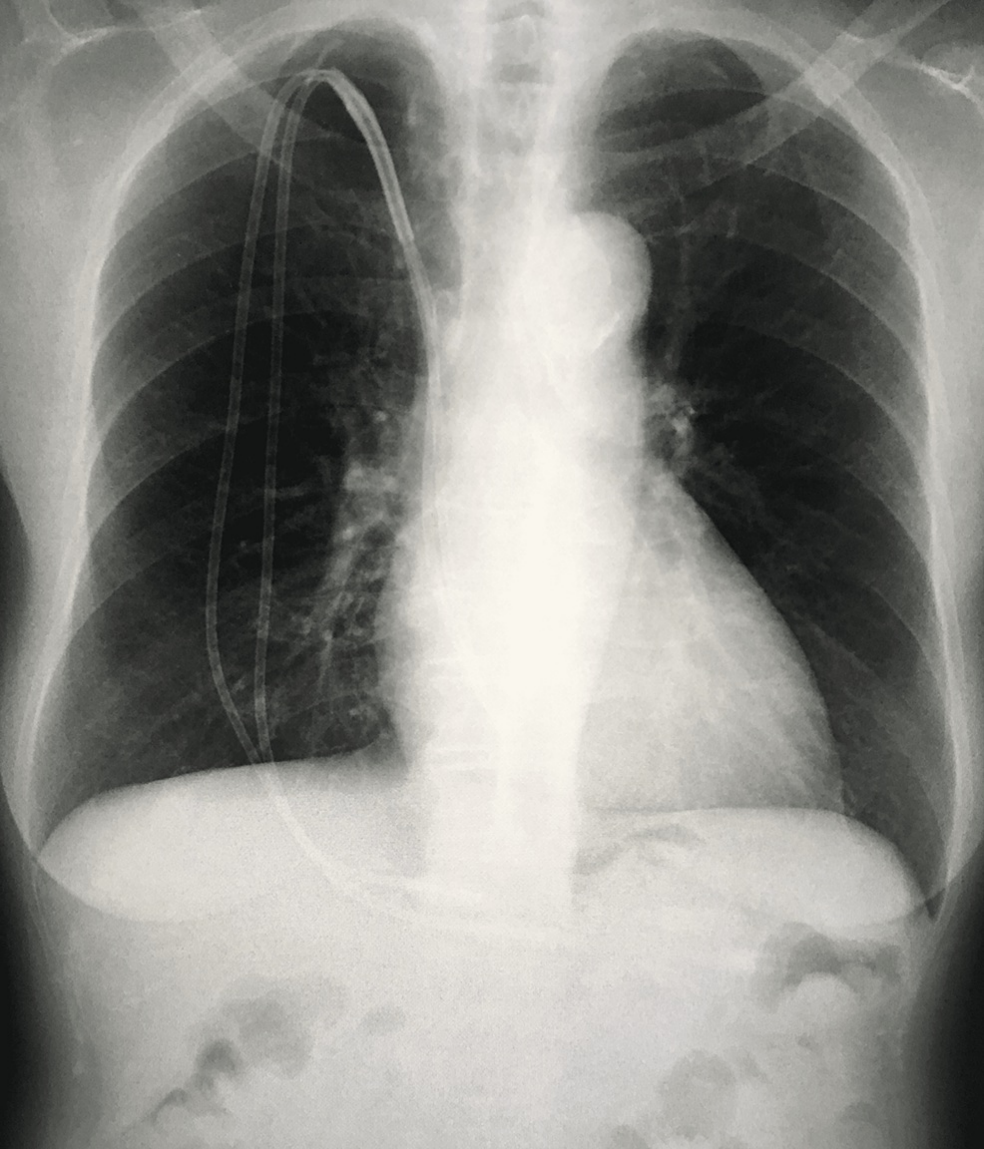
Chest radiograph showing the tunneled cuffed hemodialysis catheter inserted into the right subclavian vein.

**Figure 2:**
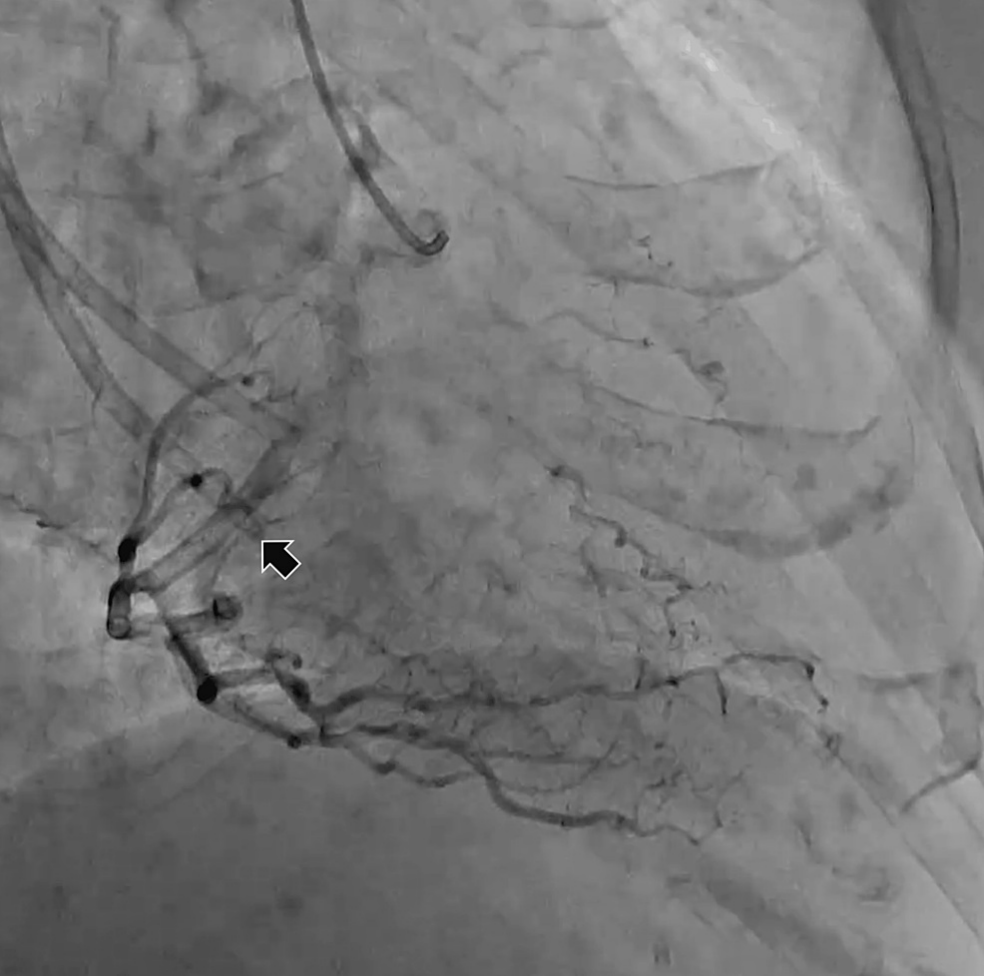
Coronary angiography showing the distal-end fracture of the hemodialysis central venous catheter (arrow).

**Figure 3:**
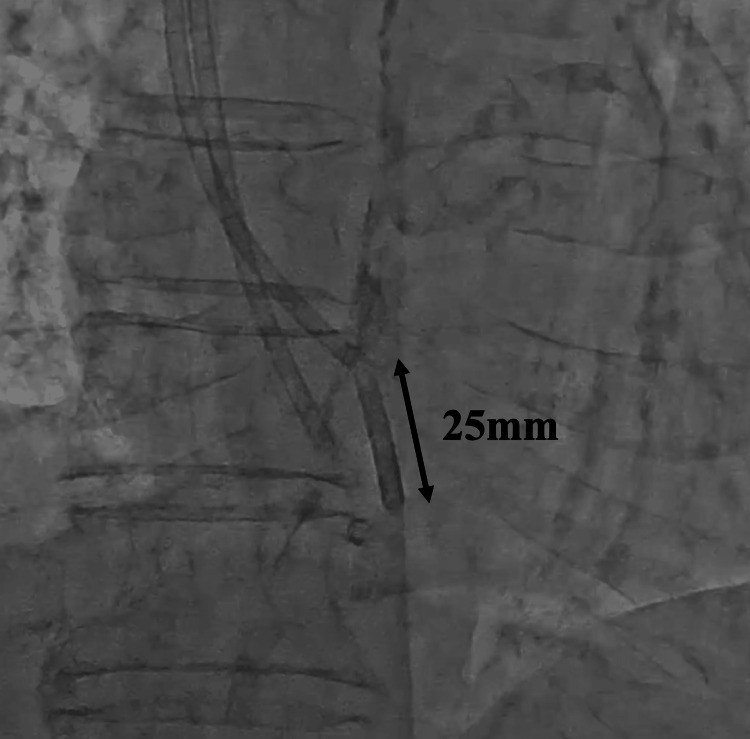
The fragmented central venous catheter (25 mm in length) in the right atrium.

A 75-year-old female, with a history of hemodialysis for five years due to diabetic nephropathy, presented to the department of cardiology with the complaint of chronic coldness and paleness of the right lower extremity with rest pain (Fontaine classification Ⅲ). For dialysis access, a tunneled hemodialysis central venous catheter (14 Fr × 36 cm, double-lumen, retrograde tunneling) was inserted into the right subclavian vein three years ago due to AVF occlusion. The patient was undergoing dialysis thrice a week without any dysfunction, and she was not complaining of chest pain or dyspnea. There were no remarkable findings from the chest X-ray (Figure 1), and an arterial duplex ultrasonography showed significant atherosclerotic calcification and no blood flow in both the anterior and posterior tibial arteries. Since there was a high likelihood of critical limb ischemia, the patient was admitted to the hospital for coronary and lower-extremity angiography. The lower-extremity angiography showed a total occlusion of the right superficial femoral artery and bilateral anterior and posterior tibial arteries. Fortunately, neither lower-extremity ulcers nor signs of infection were identified. Thus, urgent endovascular therapy was not considered essential at this point. Coronary angiography revealed no significant coronary artery stenosis; however, the distal portion of the central venous catheter was fractured (Figure 2). Additional fluoroscopy demonstrated that the fractured catheter (25 mm in length) was located at the right atrium (RA) (Figure 3).

**Figure 4:**
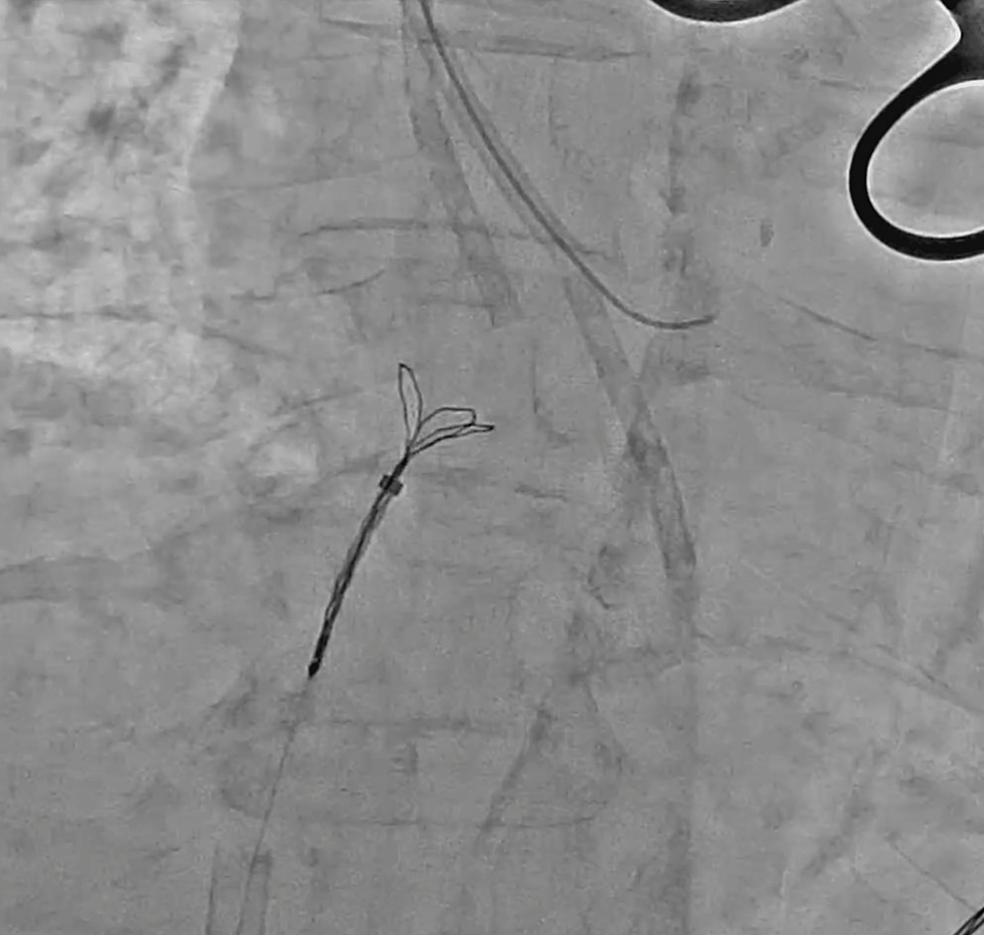
Advancing the 0.035-inch wire into the fractured part of the central venous catheter to be caught by the triple-loop snare.

**Figure 5:**
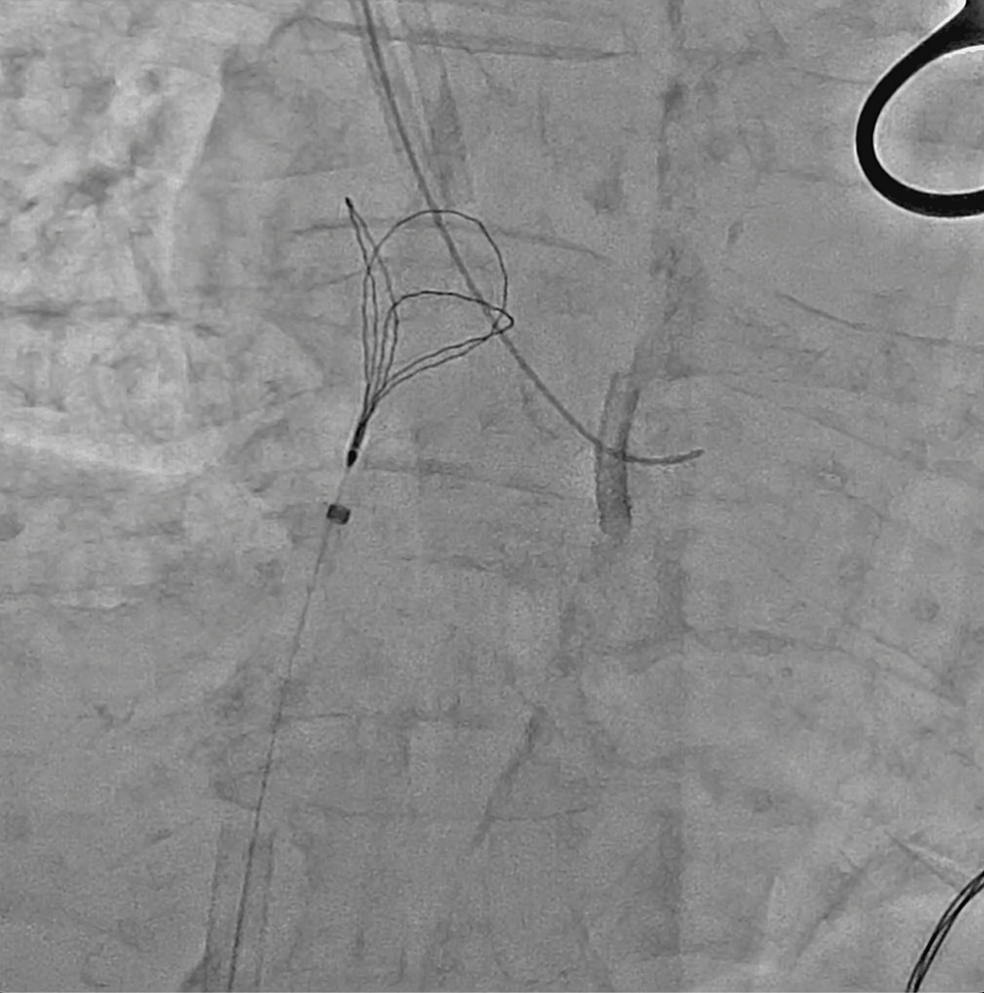
The dislodged distal portion of the central venous catheter blocked by the tricuspid valve.

**Figure 6:**
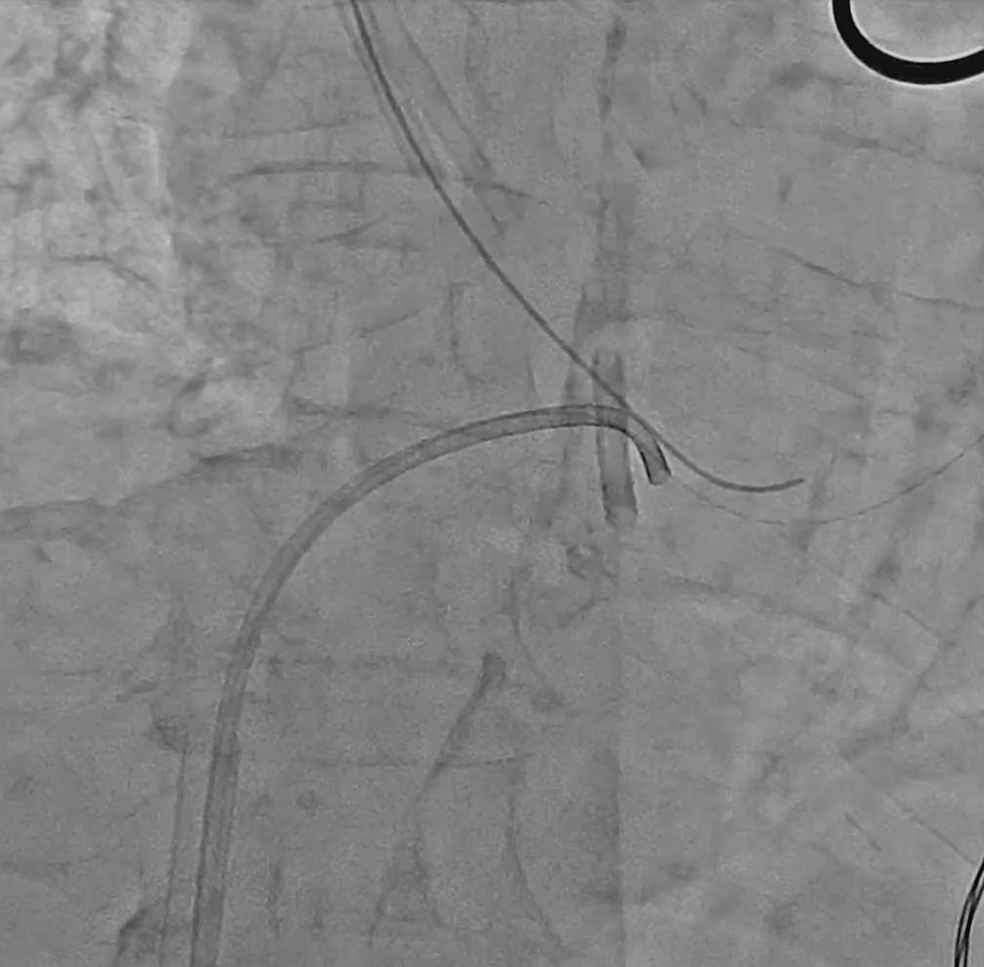
Using the 8-Fr Judkins left catheter to reposition the fractured segment of the central venous catheter.

**Figure 7:**
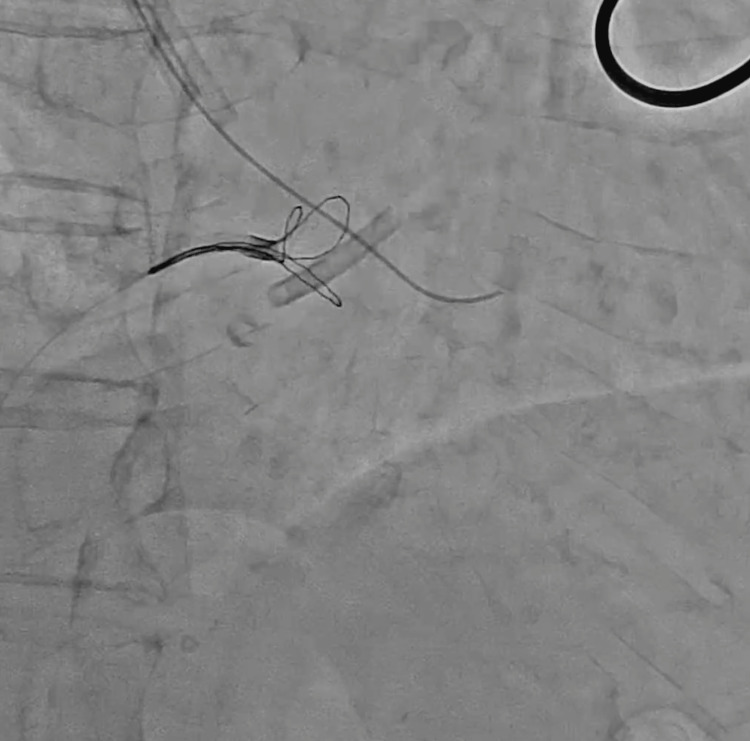
The triple-loop snare forming a loop over the dislodged central venous catheter.

**Figure 8:**
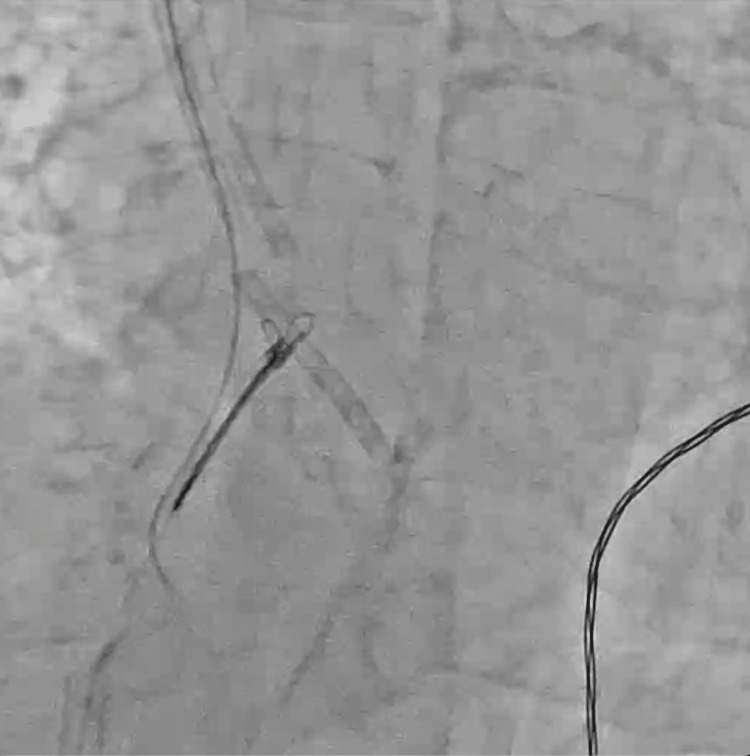
The triple-loop snare catching the fractured catheter and being pulled back into the 14-Fr sheath.

**Figure 9:**
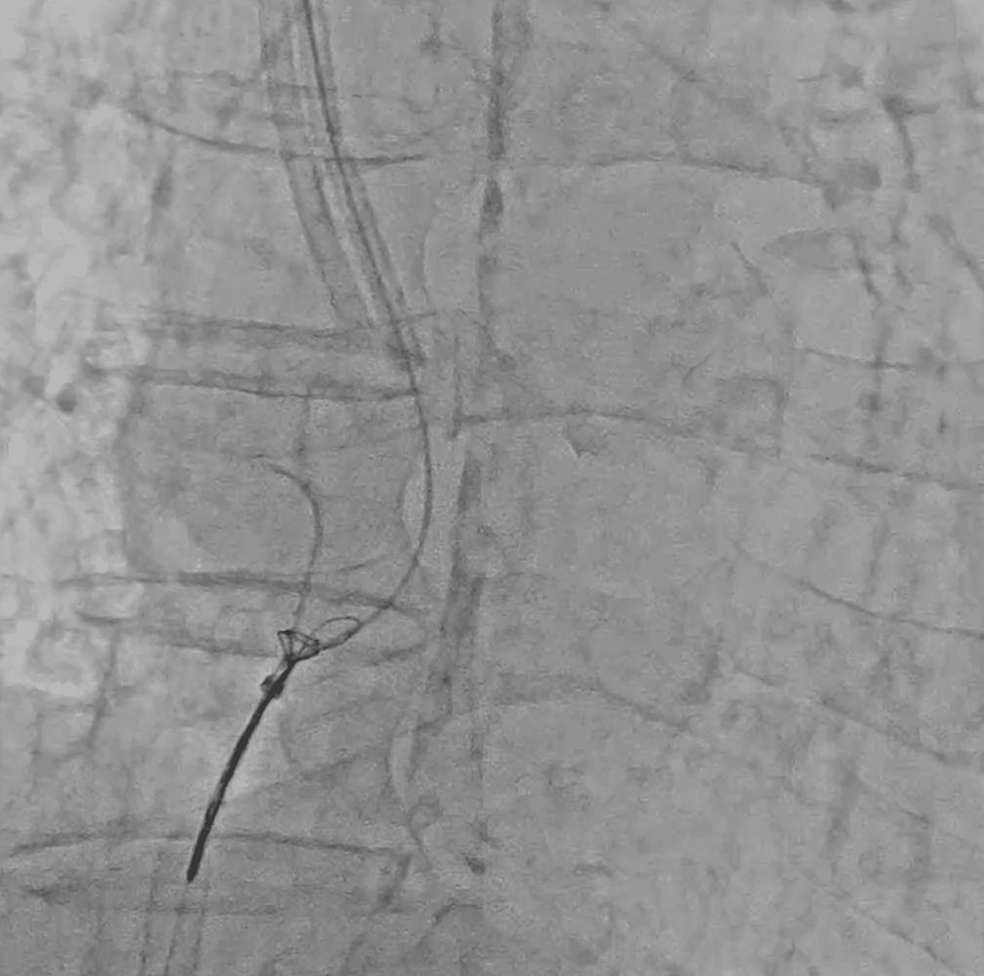
The triple-loop snare grasping the 0.035-inch wire, which was introduced into the other lumen of the catheter.

**Figure 10:**
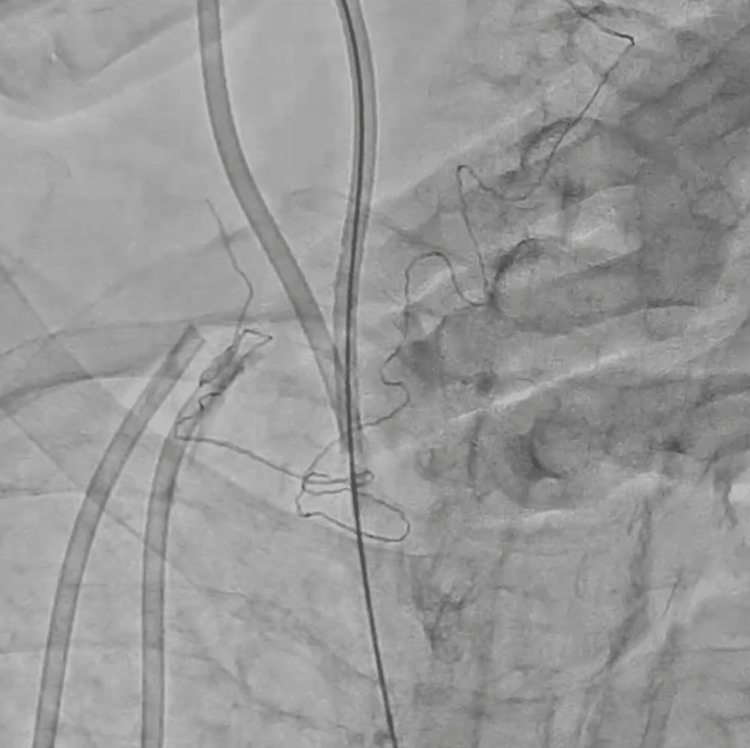
Retrieval of the whole central venous catheter from the right subclavian vein.

To retrieve the broken central venous catheter, the endovascular approach was used. For access, the right femoral vein was punctured, and a 14-Fr sheath was placed in the inferior vena cava. An EN Snare® Endovascular Snare System (Merit Medical Systems, Inc., Utah, United States) was advanced into the RA. A 0.035-inch, 150-cm wire (Terumo Corporation, Tokyo, Japan) was introduced through the lumen of the tunneled catheter, and an attempt was made to advance the wire into the fractured part of the catheter antegradely (Figure 4). However, this procedure caused the complete fragmentation of the fractured segment, which was luckily blocked by the tricuspid valve and remained in the RA, avoiding a pulmonary embolism (Figure 5). An 8-Fr 4.0 Judkins left (JL) guiding catheter (Asahi Intecc Co., Ltd, Aichi, Japan) was inserted to help reposition the fractured catheter (Figure 6), and the snare was loaded into the JL guiding catheter. Using the snare, the fractured segment was captured and safely withdrawn into the 14-Fr sheath (Figures 7-8). Despite the success of the dislodged catheter retrieval process, it was easily recognized that the distal part of the other lumen was also almost fragmented. The 0.035-inch wire was readvanced into the lumen of the venous catheter antegradely and captured by the snare to perform externalization (Figure 9). The fractured catheter was not pulled back into the 14-Fr sheath, and the hemodialysis central venous catheter was carefully removed from the right subclavian vein (Figure 10). The entire procedure of retrieving the fragmented catheter is shown in Video 1.

**Video 1:** The entire procedure of the fractured catheter retrieval.

## Discussion

As the field of endovascular intervention has made remarkable progress, inserting medical devices into patients is considered a common and safe practice. However, there is always a risk of device fracture and displacement in the body. The causes of intravascular fragment fracture include inappropriate techniques and procedures, device problems, and patient-related factors [4]. In a series of 215 cases of venous catheter embolization, pinch-off syndrome, a spontaneous catheter fracture due to the compression between the first rib and the subclavian, occurred in 40.9% of all cases, and the mechanisms were as follows: catheter damage during insertion, catheter disconnection, and proximal or distal catheter fracture [5]. In the present case, the distal portion of the catheter was almost torn off. The catheter was deeply positioned in the right subclavian vein; therefore, its distal part was possibly touching the tricuspid valve in the RA. The physical impact of valve movement was considered to have caused the catheter fracture.

Although the insertion of hemodialysis central venous catheters is a widely performed procedure in patients with ESRD, the optimal position of the catheter tip is still controversial. According to the clinical practice guideline for vascular access provided by the National Kidney Foundation's Kidney Disease Outcomes Quality Initiative, the recommended catheter tip location of tunneled cuffed catheters is the mid-RA to ensure adequate blood flow [6]. Nevertheless, a study of 993 patients with tunneled hemodialysis catheters reported that catheter tips located in the superior vena cava were significantly associated with a lower rate of catheter dysfunction (1.9%) compared to catheter tips located in the cavo-atrial junction (8%) or deep RA (11%) [7]. The appropriate position of the catheter tip varies depending on individual patients; therefore, it is essential for physicians to determine its location by fully understanding the cardiovascular structure. 

Since the first successful endovascular retrieval of a broken guidewire using bronchoscopic forceps in 1964 [8], various percutaneous transcatheter devices have been developed, such as the loop snare catheter, basket catheter, and grasping forceps [4]. Due to the advantage of flexibility, the loop snare method is a standard procedure with a high success rate in terms of retrieving dislodged catheters with accessible free ends [3,9]. In the present case, to catch the fractured segment using the snare, the 8-Fr JL was used to assist repositioning. The fractured catheter included accessible edges; thus, the whole procedure was simple and straightforward, without requiring complicated techniques. In situations that require the retrieval of intravascular fractured fragments with no accessible free ends, a pigtail catheter is essential to reposition and make a free end for snaring, which is called the two-step technique [9-11].

## Conclusions

Intravascular foreign bodies, such as those resulting from catheter fragmentation, are rarely encountered, but they can cause life-threatening complications. In the present case, early detection during the coronary angiography led to the percutaneous retrieval of the fractured catheter and prevented the patient from further adverse events, such as pulmonary embolization. It is important for healthcare providers to consider the potential risk of dislodged medical devices in their daily practice. 
